# Disease of the Peridental Membrane

**Published:** 1898-11

**Authors:** C. M. Palmer

**Affiliations:** Charles City, Iowa.


ARTICLE V.

### DISEASE OF THE PERIDENTAL MEMBRANE.

C. M. PALMER, D. D. S., CHARLES CITY, IOWA.

**Read before the Northern Iowa Dental Society, Mason .City, September, 1897.**

We are all familiar with the construction and function of the peridental membrane of a tooth, which acts as a cushion against the hardships and severe blows it is liabie to receive in the performance of tearing and grinding food.

As this membrane receives its blood and nerve supply from the same source as does tho tooth pulp, it will be easily seen that any trouble which would impair the health of that membrane would also affect the enclosed pulp, and instead of having one trouble there would be a complication.

This membrane is the medium through which the cementum and adjacent tissues receive their nourishment. It is also the organ of touch, and the only one possessed by the tooth; in fact, it can well be called the vital store-house from which, or through which, renewed forces are constantly supplied.

This membrane is subjected to various forms of disease, among which there are several distinguishable varieties of inflammation which arise from distinct causes, and require different treatment. They may be classed as: Traumatic pericementitis, or inflammation of the peridental membrane resulting from an injury; apical pericementitis, or inflammation of the peridental membrane, having its seat in the apical space and following the death of the pulp; alveolar abscess, which always has its seat in the apical space, and is the result of apical pericementitis following the death of the pulp; gingivitis, inflammation of the gingival border of the gums and neighboring border of the peridental membrane, occurring mostly from constitutional causes; calcic inflammation of the peridental membrane, and a diseased condition caused by deposits of calculus about the necks of the teeth.

We may well divide our classification of this subject into two distinct groups; one having its exciting cause in the apical space, and the other at the gingival margin.

Apical pericementitis, together with the alveolar abscess which follows, is the most painful affliction to which the teeth are liable. This trouble always begins in the apical space, and in the immediate neighborhood of the apical foramen. It never occurs during the life of the pulp of the tooth, nor does it necessarily follow immediately after the pulp's death, as it may occur months or even years after. However, no tooth with an empty pulp chamber is safe from apical pericementitis.

One instance in my own practice. I had occasion to crown a central incisor. After removing the remains of a gold filling. I proceeded to uncover the pulp preparatory to its removal, but to my surprise there was no sense of pain. As the operation advanced, the canal was opened and found to be as free from pulp tissue as though it had been removed by the usual means. Upon further investigation I found the canal to be perfectly sealed at the apex, and the chamber apparently as dry and sweet as could be made. After thoroughly sterilizing the canal, the same was properly filled and the crown set, which has been doing good service for three years. How long the tooth would have remained in its former condition cannot be known, but to all appearances it was in a perfect state of preservation, yet I am convinced that sooner or later serum would have percolated the canal and caused inflammation in the apical space.

The question has arisen that, if micro-organisms must be present before putrefaction can take place, how do they make their way into a pulp chamber when there is no external opening. However this may be, the fact remains that such decompositions do occur.

We are often called on to diagnose cases, and to decide whether they be apical pericementitis or inflammation of the dental pulp. We will find in cases of diseased pulp, the affected organ does not become tender to the touch, at least not until the inflammation has passed through the apical foramen, thus causing apical inflammation. If we but remember that the peridental membrane is the only sense organ of the tooth, we will see that if pericementitis be the trouble, the pain will be reflected definitely to the particular tooth; when in case of pulpitis we are often uncertain as to the exact location of the pain; but if we bear in mind the function of the two organs, there will be but little difficulty in rightly diagnosing these cases.

The peridental membrane being the organ of touch, therefore definitely locates in ailments, when, on the other hand, the pulp of the tooth is not an organ of touch and therefore does not definitely locate its ailments, but is prone to cause reflected pain, which may indeed be to distant parts. This seems to be characteristic of those organs that have no nerves of touch, as, for example, we find in case of hip-joint disease that pain is reflected to the knee.

Another important point is the fact that the dental pulp is especially sensitive to thermal changes, and this responsiveness is much increased in diseased conditions, when on the other hand, the peridental membrane has no such special sensitiveness.

There seems to be but one condition in which thermal changes cause marked pain in acute or chronic apical pericementitis, and that is in cases when the pulp chamber of the tooth is filled with gas, so as to cause pressure on the tissues of the apical space, when the application of heat will give rise to an expansion of the gas, which will create a greater pressure and pain, while the application of cold, in this case, gives relief.

I was once called to go seven miles to see a young lady who was suffering from a troublesome molar. The only relief afforded her was by holding ice water in her mouth. This very fact at once suggested the seat of trouble, and under the circumstances the only permanent relief that could be given was to remove the tooth, which I did, and immediately all pain ceased and sleep was afforded.

Just a word as to abscesses. Litch says an alveolar abscess results from inflammation having its seat in the apical space proceeding to the formation of pus. So we are to understand that this trouble, in its inception, is always in the apical space, no matter where it may afterwards extend.

It is quite true we may have an abscess occurring on the side of a root of a tooth, as a result of an injury and not from the death of the pulp, but such would be known as a traumatic alveolar abscess.

We find two classes of alveolar abscess, known as acute and chronic.

The latter may be formed as the result directly from chronic apical pericementitis, without having acute inflammation present at any time. Indeed, the change may have taken place so quietly and caused so little disturbance that the patient will have had no knowledge of the affected tooth even having been sore. The tooth may not be decayed at any point; its pulp may have died from any of the diseases to which it is liable, and yet may present the appearance of perfect health.

We now come to consider the diseases of the peridental membrane having their origin at the margin of the gum. This group of diseases opens to us a subject that would afford material for volumes, so we will not mention the minor divisions and subdivisions; but will speak briefly of them in a more general way.

I know of no more important duty that confronts us in the art of dentistry than to ever be on the alert to relieve our patients of any trouble that will surely cause a loss, besides giving much annoyance if allowed to remain. I am sure those of you who bear the title of our profession will agree with me that the study of these troubles is too much neglected, even in our dental colleges. To be sure, we are instructed along these lines, but it is in a general way and is apt to be covered by the term pyorrhea alveolaris, which means very little in itself, for there may be a score of byways leading to as many causes and modes of treatment.

Is it not a fact that we are daily consulted regarding some features of this trouble, and how often we do not give our patients all they are entitled to! Many times our only excuse is that it is not a desirable operation to perform, and again it may be owing to the small fee we usually receive for length of service rendered.

Not long ago a lady came to me for advice, and upon examination I found the lower incisors to be very loose, and especially were the centers so badly affected that they were giving much trouble and required removal. Apparently those teeth were perfectly sound, but they were completely surrounded by a calcic deposit, which had caused the absorption of the alveolus to such an extent that a calcic deposit had formed quite the full length of the roots. This lady had been under the care of one dentist for fourteen years, and at this time had been told that she must lose all her teeth.

There is little doubt that simple gingivitis is often the starting point of the more grave diseases of the peridental membrane, and may begin from neglect in properly cleansing the teeth and oral cavity, and in time will cause an eversion of the gums, which favors the lodgment of calculus, and a little later inflammation of the peridental membrane.

Calcic inflammation is one of the most grave diseases, not that it is so very difficult to manage when rightly understood, but from the great number of cases that occur and its destructive character. My observation teaches that it is causing the loss of more teeth than is caries.

We have the two forms of calcic formation, viz.: serumal and salivary.

The latter is derived from the saliva that exudes from the tissues in a state of disease, and is uniformly deposited under the free margin of the gums, but not beneath them. When a slight deposit has once taken place it becomes an irritant, which will in itself perpetuate the disease, as it seems to possess peculiar irritating qualities which keeps the adjacent gums in a state of chronic inflammation which may continue many years, but finally we have a deepening and widening of the disease until the peridental membrane becomes affected to such an extent that it is finally destroyed, exposing the necks of the teeth, and as fast as the membrane is detached from the roots, the rim of the alveolar wall is absorbed and the gums recede with it.

As these conditions continue, the peridental membrane becomes more diseased, pockets are formed, and we have both forms of calculus taking place; all the symptoms become more aggravated, and there is a continual flow of pus from the necks of the diseased teeth, which gradually become loosened and require removal.

It has been the aim of this brief paper to call attention anew to some of the many diseases to which the surrounding tooth structure is heir, and to inquire whether or not it is not a fact that we too often neglect making a careful study of these cases and treat accordingly, but extract many teeth which might be made to do good service for many years.—*Items of Interest.*

